# Place of Next Meeting

**Published:** 1867-06

**Authors:** 


### PLACE OF NEXT MEETING.

The subject of the next place of meeting was taken up, by Dr. Hammer moving that St. Louis be designated as the next place.

The subject was finally disposed of, by choosing Washington City as the place of meeting, and requiring the Committee of Arrangements to avoid any entertainments that will interfere with the business of the Association or of the Sections.

